# Evaluation of the reliability and internal consistency of the child eating behavior questionnaire (CEBQ) in spanish schoolchildren and its association with obesity

**DOI:** 10.1002/brb3.70343

**Published:** 2025-02-19

**Authors:** Andrea Calderón García, Roberto Pedrero‐Tomé, Ana Alaminos‐Torres, Noemí López‐Ejeda, María Sánchez Álvarez, Consuelo Prado Martínez, Jesús Román Martínez Álvarez, María Dolores Marrodán Serrano

**Affiliations:** ^1^ Research Group EPINUT (Nutritional Epidemiology) Faculty of Medicine Complutense University of Madrid Madrid Spain; ^2^ Department of Pharmacy and Nutrition, Faculty of Biomedical Sciences European University of Madrid, UEM Villaviciosa de Odón Madrid Spain; ^3^ Department of Biodiversity, Ecology and Evolution, Faculty of Biology Complutense University of Madrid Madrid Spain; ^4^ Faculty of Health Sciences and Faculty of Humanities and Social Sciences of the International University Isabel I of Burgos Burgos Spain; ^5^ Department of Biology, Faculty of Sciences Universidad Autónoma de Madrid Madrid Spain

**Keywords:** appetite, eating behavior, pediatric obesity, satiety response

## Abstract

**Introduction:**

Certain behavioral traits increase the risk of obesity at early ages. Exploring patterns of eating behavior in schoolchildren using tools like the Children's Eating Behavior Questionnaire (CEBQ) is crucial for obesity prevention and intervention. Before this study, the CEBQ had been validated in various languages, including Spanish, but only in preschool‐aged children, despite its previous use in various studies in the Spanish language and population. The objective is to assess the reliability and internal consistency of the CEBQ questionnaire applied to a sample of Spanish schoolchildren and to explore the association between eating behavior assessed through the CEBQ and nutritional status based on BMI.

**Methods:**

Parents or guardians of 283 Spanish schoolchildren aged 6–16 years completed the Spanish version of the CEBQ questionnaire. Factor analyses were conducted on all CEBQ items, and differences between genders and age groups were examined. Correlations between children's BMI *Z*‐scores and eating behaviors were analyzed through linear regression.

**Results:**

The factor analysis revealed that the CEBQ is suitable in its original structure of eight subscales translated into Spanish, demonstrating adequate reliability and acceptable correlations between subscales. Gender appears to have minimal influence on eating behavior. However, with increasing age, schoolchildren tend to eat more quickly and are less fussy about food (SE [slowness in eating] [*p* = 0.012] and FF [food fussiness] [*p* = 0.012] decrease). Positive associations were found between BMI Z‐scores and all pro‐eating dimension subscales, while negative associations were identified with all antieating dimension subscales (*p* < 0.05) except EUE (emotional undereating) (*p* = 0.106).

**Conclusions:**

The CEBQ is a valid psychometric tool that can be reliably used to assess eating behavior characteristics in Spanish schoolchildren aged 6–16 years.

## Graphical abstract

This study evaluates the reliability of the Spanish version of the Children's Eating Behaviour Questionnaire (CEBQ) in children aged 6–16. Results show an association between pro‐eating behaviors and higher BMI, emphasising the CEBQ's role in identifying traits linked to obesity risk in pediatric populations.

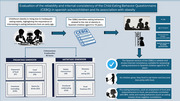

**Abbreviations**
BMI: body mass index
CEBQ: Children's Eating Behavior Questionnaire
CFA: confirmatory factor analyses
DD: desire for drinks
EF: enjoyment of food
EOE: emotional overeating
EUE: emotional undereating
FF: food fussiness
FR: food responsiveness
SE: slowness in eating
SR: satiety responsiveness

## 1 Introduction

Overweight and obesity in children and adolescents have experienced a dramatic increase worldwide (Di Cesare et al. 2019). The Childhood Obesity Surveillance Initiative (COSI) by the World Health Organization (2022), which includes data from the ALADINO study (ALimentación, Actividad física, Desarrollo INfantil y Obesidad) published in 2019, places Spain fourth in the European ranking for excess weight (overweight + obesity) with a prevalence of 38.4% in boys and 39.3% in girls aged 6–9 years. Similarly, the cross‐sectional PASOS study in the Spanish population aged 8–16 years reports a prevalence of excess weight of 33.4% (21.6% overweight in both genders and 11.8% obesity [15.3% in boys and 8.4% in girls]) (Gómez et al. 2023).

The development of overweight and obesity is complex and multifactorial, influenced by genetic, psychosocial, and environmental factors, with diet being a primary contributor, as emerging dietary patterns show a progressive decrease in the consumption of fruits, vegetables, and greens (López‐Sobaler et al. 2021). In addition, there is an increase in processed foods that contribute significantly to the intake of sugar, saturated fats, refined flour, and salt (Juul et al. 2018). According to the COSI study, only 43% of schoolchildren in the European region consume fruit daily (37% in the case of Spain). Vegetable consumption is also low, observed in only 34% of European schoolchildren (only 13% in Spain). On the other hand, the SENDO study (Child monitoring for optimal development in Spanish; da Rocha et al. 2021) showed that in Spanish children aged 3–5 years, 32.2% of the total energy intake came from ultra‐processed foods.

The choice of foods is influenced by both external and intrinsic elements of the individual. Socioeconomic and cultural circumstances condition dietary preferences, but so do genetic and perinatal factors (Scaglioni et al. 2018). The review conducted by Dantas and da Silva (2019) demonstrates that in childhood, eating behavior is highly dependent on the family environment, takes shape in the early years of life, and often persists into adolescence, promoting the development of overweight when dietary practices are incorrect or unhealthy. However, the aforementioned authors also highlight, based on the literature consulted, that the effect of an obesogenic environment, culturally unfavorable conditions, or negligent parents can be counteracted through interventions at the individual, family, and collective levels (Dantas and da Silva 2019). Likewise, Dantas and da Silva (2019) emphasize that understanding and modifying eating behavior require action on different levels: physical, psychological, familial, cultural, and media‐related.

The review conducted by Llewellyn and Fildes (2017) on the theory of behavioral susceptibility and the role of appetite in the genetic risk of obesity, initially proposed by Carnell and Wardle (2008), has been crucial in establishing the relationship between eating behavior and the predisposition to gain weight. It seems evident that the preference for certain foods and behaviors related to eating modulates the genetic risk of developing obesity. Some studies, such as the one conducted by Gross et al. (2016), found differences in the behavioral phenotypes of girls with obesity and severe obesity, showing that the latter had a significantly lower satiety responsiveness (SR) than the former. Other characteristics, such as enjoyment of food (EF) or slowness in eating (SE), were also higher in patients with morbid obesity. Precisely, the ability to enjoy food or motivation to eat is associated with a higher number of daily meals and the habit of snacking, which in turn promotes obesity (Rudy et al. 2018). Emotional aspects also positively or negatively influence the amount of food that children ingest and, consequently, their weight status (Buja et al. 2022).

As mentioned, scientific literature suggests that childhood obesity results from an interaction between genetic susceptibility to overeating and exposure to an obesogenic food environment. However, this relationship does not appear to be deterministic, as certain actions at the individual, family, or community level can act as a mechanism to curb or mitigate the development of obesity (Dantas and da Silva 2019). For this reason, it is important to examine eating behavior patterns in early childhood, as this period (infancy to 6–7 years) is crucial for the development of eating behaviors (Gahagan 2012). Tools such as the Children's Eating Behavior Questionnaire (CEBQ) (Wardle et al. 2001) have proven to be useful in this regard.

For this reason, it is important to explore patterns of eating behavior in the early stages of life, and tools such as the CEBQ (Wardle et al. 2001) have proven their utility. This multidimensional psychometric test identifies behavioral phenotypes or “appetitive traits” that may favor food intake (EF, food responsiveness [FR], eating in the absence of hunger, low SR) or decrease it (SE, food fussiness [FF], high SR, among others). These traits show quantitative associations with weight and various indicators of fat quantity and distribution (Croker et al. 2011; Calderón García et al. 2023).

The CEBQ has been used in different populations of preschool and school‐age children. The first study that developed and validated the test was conducted by Wardle et al. (2001) in the United Kingdom. Subsequently, it has been translated and/or validated in different languages and applied to Portuguese populations (Viana et al. 2008), Dutch populations (Sleddens et al. 2008), Swedish populations (Svensson et al. 2011; Somaraki et al. 2022), and Polish populations (Malczyk et al. 2022). It has also been used in Australian boys and girls (Mallan et al. 2013), American populations (Domoff et al. 2015), Icelandic children aged 8–12 years (Njardvik et al. 2018), Chinese children aged 9–12 years (Gebru et al. 2021), Ethiopian 6‐year‐olds (Gebru et al. 2021), and Thai children aged 9–12 years (Sirirassamee and Hunchangsith 2016). Translated into Spanish, the CEBQ has shown high reliability and internal validity when applied to a sample of Chilean children aged 2–4 years (Henríquez Konings et al. 2018) , Mexican children aged 1–3 years (Rueda‐Sánchez et al. 2022), and Spanish children aged 3–6 years (Jimeno‐Martínez et al. 2022).

The objective of the present study is, first and foremost, to assess the reliability and internal consistency of the CEBQ questionnaire applied to a sample of Spanish boys and girls aged between 6 and 16 years. Second, our aim is to explore the association between eating behavior assessed by the CEBQ and nutritional status expressed through the Body Mass Index (BMI), taking into account gender and age.

## 2 Methods

### 2.1 Participants

The study participants consisted of 283 students aged between 6 and 16 years (33.21% female), recruited between 2019 and 2021 from schools and sports facilities in the Community of Madrid (Spain). Children of all weight categories, namely normal weight, overweight, and obesity, were included in the study. The sample size meets the requirements established by Lloret‐Segura et al. (2014), fulfilling the recommended participant‐to‐item ratio of at least 5:1, which ensures robust and reliable statistical results in factorial analysis. No exclusion criteria have been defined, except that they meet the specified characteristics, as the objective is to understand the real situation of these schoolchildren. The data used in the study are anonymized and disaggregated, ensuring that the collected information does not allow the identification of any individual. The study was approved by the Ethics Committee of the Autonomous University of Madrid (CEI‐91‐1699). Furthermore, work was conducted in accordance with the bioethical principles of the most recent version of the Declaration of Helsinki (World Medical Association 2013), and it was considered mandatory to have the informed consent of the mothers, fathers, and/or guardians of the students.

### 2.2 Procedure

The demographic characteristics of the participants and information regarding the age, gender, education, and occupation of the parents were collected through a parent‐reported questionnaire. Each participant underwent an anthropometric assessment, and their parents or guardians completed the CEBQ questionnaire (Wardle et al. 2001).

### 2.3 Anthropometric Study

Each participant underwent an anthropometric assessment following the International Biological Program (IBP) protocol (Weiner and Lourie 1981). Bilateral measurements were taken on the left side, considered the best reflection of biological conditions, assuming that the majority of the human population is right‐handed. All measurements were performed using certified and properly calibrated anthropometric equipment to ensure standardization. Direct measurements were taken individually in isolated spaces provided by the schools or sports centers, with the aim of safeguarding the participants' privacy. Each participant underwent an anthropometric assessment conducted by dietitians–nutritionists, and biologists with expertise in anthropometry.

### 2.4 Administration of the CEBQ

The CEBQ (Wardle et al. 2001) is a psychometric test that allows for an understanding of the eating behavior of children and adolescents, assessing eight dimensions: EF (four items), FR (five items), emotional overeating (EOE; four items), desire for drinks (DD; three items), SR (five items), SE (four items), emotional undereating (EUE; four items), and FF (six items). The first four dimensions have a positive or pro‐intake focus, while the last four relate to food avoidance or anti‐intake.

The conceptual meaning of each dimension or subscale is outlined in Table 1. These eight dimensions are categorized into two groups: the “pro‐intake” dimensions (EF, FR, EOE, and DD) and the “anti‐intake” dimensions (SR, SE, EUE, and FF).

**TABLE 1:** Definition of each subscale of the CEBQ.

Pro‐intake dimension	Definition
1. Enjoyment of food (EF)	Condition positively associated with hunger sensation, desire to eat, and pleasure from food
2. Food responsiveness (FR)	Susceptibility to preferring foods with better organoleptic properties in habitual contexts
3. Emotional overeating (EOE)	Tendency to increase intake in negative emotional contexts
4. Desire for drinks (DD)	Desire for drinks and tendency to have sugary beverages readily available
Anti‐intake dimension	Definition
5. Satiety responsiveness (SR)	Decrease in hunger sensation caused by the consumption of food
6. Slowness in eating (SE)	Tendency to eat more slowly during a meal and extend its duration
7. Emotional undereating (EUE)	Tendency to decrease intake in negative emotional contexts
8. Food fussiness (FF)	Conditioned demand that limits the range of accepted food products

*Source*: González and Santos Martínez (2011).

The CEBQ consists of 35 items, each scored on a Likert‐type scale ranging from 1 to 5, where 1 = never, 2 = rarely, 3 = sometimes, 4 = often, and 5 = always, based on the frequency of the behavior. The interpretation of the scores is as follows: higher scores indicate more frequent or intense behaviors, while lower scores reflect less frequent behaviors. In addition, five items are reverse‐scored (Questions 3, 4, 10, 16, and 32), as detailed in Table S1. This scoring system enables the assessment of each eating behavior trait, with the scores corresponding to the intensity of each behavior.

The version of the CEBQ used in this study was adapted from versions employed in previous research conducted in Spanish and Chilean populations (González and Santos Martínez 2011; Sánchez et al. 2016; Jimeno‐Martínez et al. 2022). Following the recommendations of Tsang et al. (2017), a back‐translation into English of the Spanish version was performed to verify its accuracy. In cases of discrepancies between the versions, a detailed analysis was conducted to select the option that most accurately reflected the original meaning of the questionnaire. A thorough review was then carried out to identify any elements that could cause confusion or lead to biased responses. Subsequently, a pilot test was administered with a sample of 10 individuals from the target population to identify potential ambiguities and make necessary corrections. The final version of the questionnaire is provided in the supplementary material (Table S1).

### 2.5 Statistical Procedures

The general characteristics of the study sample were analyzed using mean and standard deviation (SD) for continuous variables and *N* and percentage for categorical variables. Depending on the normality of the variables, ANOVA, Mann–Whitney *U*, or Kruskal–Wallis tests were conducted to compare the mean scores of each CEBQ subscale according to gender, age, nutritional categories, and family factors.

For the prevalence analysis, the sample was stratified by gender and age. Nutritional categories were established based on age‐and‐gender‐adjusted BMI using the reference values published by Cole et al. (2000, 2007). The BMI values were calculated by transforming each student's BMI into a standardized *Z*‐score, adjusting for age and gender. Overweight was defined as a BMI > +1 SD, obesity as a BMI > +2 SD, and thinness associated with malnutrition as a BMI < −2 SD. This approach ensures that the BMI values are appropriately adjusted for the participant's age and gender, following the Cole reference standards.

To verify the structure of the Spanish version of the questionnaire, a confirmatory factor analysis (CFA) was conducted on the original 8‐factor model, following the approach of Jimeno‐Martínez et al. (2022). Before this, sample adequacy was assessed using the Kaiser–Meyer–Olkin (KMO) test, which evaluates the suitability of the data for factor analysis. The KMO value ranges from 0 to 1, with values above 0.60 generally considered acceptable for proceeding with the analysis. Values closer to 1 indicate better suitability, with values greater than 0.90 considered excellent, between 0.80 and 0.90 considered good, and between 0.70 and 0.80 considered acceptable. Values below 0.60 suggest inadequate data for factor analysis. In addition, Bartlett's test of sphericity was performed to assess the hypothesis that the correlation matrix is an identity matrix. A significant result from this test indicates that the variables are sufficiently correlated to proceed with factor analysis. Finally, Varimax rotation was applied to obtain the component matrix and classify each of the 35 items based on the highest factor loading in its corresponding scale. A factor loading threshold of 0.4 was set as an appropriate limit for CFA (Svensson et al. 2011).

The internal consistency of the CEBQ questionnaire was evaluated to assess the reliability of the test in the present sample. The internal consistency of the eight subscales of the CEBQ questionnaire and reliability estimates were then determined using Cronbach's alpha, with values > 0.70 considered acceptable, although values close to 0.60 are also deemed suitable by some authors (Nunally 1978).

To assess the suitability of the CEBQ for the present sample, statistical fit indices recommended by Hu and Bentler (1999) , such as RMSEA, comparative fit index (CFI), Tucker–Lewis index (TLI), and standardized root mean square residual (SRMR), were used, following methodologies similar to those employed in studies exploring the validity of the CEBQ across different populations and geographical areas (Al‐Hamad et al. 2021; Jimeno‐Martínez et al. 2022; Warkentin et al. 2022). To assess the relationships between scales, a correlation analysis was conducted, and a hierarchical multiple linear regression model was applied to examine the association between the BMI *Z*‐scores of the students (continuous dependent variable) and each eating behavior subscale (independent variables), controlling for gender and age (grouped into 6–10 years, prepubertal, and 11–16 years, pubertal). The age division into prepubertal and pubertal allows capturing the differences in biological and behavioral development between childhood and adolescence, as supported by previous literature (Hernández et al. 2008). Statistical analysis was performed using SPSS Statistics 21.0 and R 4.1.2 software. Statistical significance was considered when *p* < 0.05.

## 3 Results

### 3.1 Assessment of Internal Consistency and Reliability: CFA

The KMO test yielded a value of 0.872, which indicates that the data is highly suitable for conducting factor analysis. Likewise, Bartlett's test of sphericity was significant (*χ* ^2^ = 5880.118, df = 595, *p* < 0.001), confirming the existence of sufficient correlations among the variables to justify the factorial analysis.

Subsequently, the number of factors in the questionnaire and their factor loading were analyzed through CFA on the original 8‐factor model. The eight factors with eigenvalues greater than 1 explained 68.83% of the total variance, with FR (identified through the maximum likelihood method after conducting parallel analysis) being especially prominent. Varimax rotation was then applied to obtain the rotated component matrix and classify each of the 35 items based on the highest value in its corresponding scale.

The model fit indices further confirmed the adequacy of the proposed 8‐factor structure. The RMSEA was 0.066, indicating a good fit, with a 90% confidence interval of [0.060, 0.072]. In addition, CFI was 0.911, the TLI reached 0.914, and the SRMR was 0.072. These values meet established thresholds for acceptable fit and suggest that the hypothesized structure appropriately explains the observed data.

Internal consistency was adequate (Cronbach's alpha above 0.70) for all factors except for the EF subscale, where the value was slightly lower but still above 0.60, a value also considered acceptable. Therefore, it can be stated that the CEBQ questionnaire exhibits relatively high internal consistency. The unweighted mean scores of the factors (±SD) and internal reliability estimates (Cronbach's alpha) for each CEBQ factor are presented in Table 2.

**TABLE 2:** Mean score and internal consistency of the CEBQ in the analyzed sample (*n* = 283).

Dimension	Subscale	Mean (SD)	Cronbach's alpha
Pro‐intake	1. Enjoyment of food (EF)	3.90 (0.25)	0.631
	2. Food responsiveness (FR)	2.38 (0.99)	0.879
	3. Emotional overeating (EOE)	2.15 (0.69)	0.814
	4. Desire for drinks (DD)	2.39 (0.68)	0.842
Anti‐intake	5. Satiety responsiveness (SR)	2.42 (0.66)	0.815
	6. Slowness in eating (SE)	2.47 (0.57)	0.839
	7. Emotional undereating (EUE)	2.43 (0.47)	0.768
	8. Food fussiness (FF)	2.69 (0.80)	0.839

In this final model, the 35 items were loaded onto their respective eight factors with factor loadings greater than 0.40 (*p *< 0.001), except for four items that showed cross‐loadings. One of these items was present in subscale EF, another in subscale SR, and the other two in subscale EOE, with their loadings being higher in subscale FR in all cases (Table 3). Following the methodology of previous literature, items with cross‐loadings were kept in the original CEBQ subscale to harmonize results and facilitate comparison (Jimeno‐Martínez et al. 2022).

**TABLE 3:** Factor loadings for the items of the CEBQ questionnaire from the confirmatory factor analysis.

Factor	Percentage of variance	Scale	Factor loading	Cronbach's alpha
**Factor 1**	24.94			0.879
**Subscale 2: Food responsiveness (FR)**		12. My child is always asking for food.	0.757	
		14. If allowed, my child would eat too much.	0.661	
		19. Given the opportunity, he would eat most of the time.	0.786	
		28. Even if my child is full, he/she always has room for his/her favorite food.	0.630	
		34. If I give him/her the chance, my child always has food in his/her mouth.	0.661	
**Factor 2**	16.91			0.872
**Subscale 8: Food fussiness (FF)**		7. My child initially refuses new foods	0.855	
		10. Enjoys tasting new foods	0.769	
		16. Enjoys a variety of foods	0.585	
		24. My child is hard to please with meals	0.511	
		32. My child is interested in trying foods he/she has not tasted before	0.834	
		33. My child decides he/she doesn't like a food even without trying it	0.832	
**Factor 3**	7.81			0.839
**Subscale 6: Slowness in eating (SE)**		8. Eats slowly	0.791	
		18. Takes more than 30 min to finish a meal	0.779	
		35. My child eats more and more slowly while the meal	0.675	
		4. My child finishes his/her meal quickly	0.760	
**Factor 4**	5.55			0.768
**Subscale 7: Emotional undereating (EUE)**		9. Eats less when he/she is angry	0.804	
		11. Eats less when he/she is tired	0.753	
		25. Eats less when he/she is upset	0.760	
		23. Eats more when he/she is happy	0.464	
**Factor 5**	4.75			0.631
**Subscale 1: Enjoyment of food (EF)**		1. My child loves food	0.646	
		5. Is interested in food	0.685	
		20. My child waits to eat at established mealtimes (0.609 in factor 1)	0.347	
		22. My child enjoys eating	0.603	
**Factor 6**	3.22			0.815
**Subscale 5: Satiety responsiveness (SR)**		3. My child has a good appetite (0.568 in factor 1)	0.326	
		17. My child leaves food on the plate when finishing a meal	0.475	
		21. My child gets full before finishing his/her meal	0.744	
		26. My child gets full easily	0.685	
		30. If my child has had something to drink before, he/she doesn't feel like eating	0.647	
**Factor 7**	2.97			0.842
**Subscale 4: Desire for drinks (DD)**		29. If given the opportunity, my child would constantly drink throughout the day	0.842	
		31. If I give him/her the chance, he/she would always be drinking something	0.820	
		6. My child asks for liquids all the time	0.814	
**Factor 8**	2.74			0.814
**Subscale 3: Emotional overeating (EOE)**		2. My child eats more when he/she is worried	0.796	
		15. Eats more when he/she is anxious	0.669	
		13. My child eats more when he/she is upset/bored (0.691 in factor 1)	0.108	
		27. My child eats more when he/she has nothing to do (0.755 in factor 1)	0.146	

*Note*: Confirmatory factor analysis based on factor loading and items grouped by scale. Internal consistency of the scales was calculated using Cronbach's alpha. Additional factor loadings are provided for items that loaded on more than one scale.

Subsequently, the correlation between the subscales of the CEBQ was evaluated. The results of the correlation analysis among the scores of the different scales revealed a significant association in most cases. The strongest positive correlation was found between the EOE and FR subscales, as well as between SR and SE. Conversely, negative correlations were identified between opposing scales, particularly between EF and SR and FF and EF (Table S2).

### 3.2 The General Characteristics of the Sample

Table 4 displays the distribution of the sample by gender and age, along with the nutritional status determined by BMI and the parents' level of education and employment.

**TABLE 4:** Sociodemographic and anthropometric characteristics of the participants.

	% (*N*)
Gender	
Boys	66.69 (189)
Girls	33.32 (94)
Age	
6	5.70 (16)
7	8.80 (25)
8	12.00 (34)
9	11.70 (33)
10	16.60 (47)
11	13.80 (39)
12	11.30 (32)
13	10.20 (29)
14	5.30 (15)
15	3.20 (9)
16	1.40 (4)
BMI^a^	
Normal weight	65.00 (184)
Overweight	24.00 (14.80)
Obesity	31.80 (90)
Mother education	
Primary level	27.70 (78)
Secondary level	32.70 (93)
Tertiary or university level	39.60 (112)
Father education	
Primary level	39.50 (111)
Secondary level	33.10 (94)
Tertiary or university level	27.90 (78)
Parental employment status	
One or both parents unemployed	22.40 (64)
Both parents employed	77.60 (220)

Abbreviation: BMI, body mass index.

^a^ Applying the BMI cut‐off points proposed by Cole et al. (2000, 2007).

### 3.3 Differences in CEBQ Based on Gender, Age Group, and Nutritional Status of Schoolchildren

In Table 5, the results of the average scores in each of the eight CEBQ subscales by gender and age group are presented. Gender seems to have little influence on eating behaviors as there were only differences in the DD subscale where males obtained higher scores. Regarding age, SE and FF decreased in the 11–16 age group compared to the 6–10 age group, meaning older schoolchildren are faster and less fussy eaters.

**TABLE 5:** Gender and age group differences in eating behavior according to CEBQ.

		Gender			Age group			Total
	Subscale CEBQ	Male (mean ± SD)	Female (*X *± SD)	*p*	6–10 years (mean ± SD)	11–16 years (mean ± SD)	*p*	
Pro‐intake	EF	3.90 ± 0.64	3.88 ± 0.68	0.791	3.85 ± 0.67	3.94 ± 0.63	0.251	3.89 ± 0.66
	FR	2.41 ± 1.01	2.31 ± 0.96	0.582	2.38 ± 0.96	2.37 ± 1.04	0.662	2.38 ± 0.99
	EOE	2.18 ± 0.91	2.03 ± 0.90	0.332	2.03 ± 0.82	2.28 ± 0.99	0.045^*^	2.15 ± 0.91
	DD	2.47 ± 0.96	2.24 ± 0.86	0.048^*^	2.35 ± 0.88	2.43 ± 0.99	0.650	2.39 ± 0.93
Anti‐intake	SR	2.40 ± 0.72	2.46 ± 0.72	0.464	2.48 ± 0.69	2.35 ± 0.75	0.100	2.42 ± 0.72
	SE	2.41 ± 0.95	2.56 ± 0.91	0.167	2.58 ± 0.92	2.33 ± 0.95	0.012^*^	2.47 ± 0.95
	EUE	2.40 ± 0.88	2.47 ± 0.90	0.617	2.39 ± 0.82	2.45 ± 0.96	0.671	2.42 ± 0.88
	EF	2.73 ± 0.89	2.60 ± 0.97	0.108	2.80 ± 0.88	2.54 ± 0.93	0.012^*^	2.68 ± 0.92

*Note*: Mean comparison using the Mann–Whitney *U* test based on gender and age group.

Abbreviations: CEBQ‐DD, desire for drinks; CEBQ‐EF, enjoyment of food; CEBQ‐EOE, emotional overeating; CEBQ‐EUE, emotional undereating; CEBQ‐FF, food fussiness; CEBQ‐FR, food responsiveness; CEBQ‐SE, slowness in eating; CEBQ‐SR, satiety responsiveness; SD, standard deviation; *X*, mean.

* *p* < 0.05.

On the other hand, regression analyses (Table 6) revealed significant associations between BMI *Z*‐scores and six out of the eight eating behavior subscales when controlled for gender and age. Positive correlations were identified with the scores of the four pro‐intake subscales, meaning that for each additional point, the BMI *Z*‐score increased by 0.19 (EF), 0.25 (FR), 0.21 (EOE), and 0.12 points (DD). Regarding the anti‐intake subscales, negative correlations were found with SR and SE, so that for each additional point, it reduced the BMI *Z*‐score by 0.27 and 0.32, respectively.

**TABLE 6:** Linear regression analysis for BMI *Z*‐score on CEBQ subscales.

Independent variables	*β*	Exp (*β*)	CI	*p*	*R* ^2^
CEBQ‐EF	0.320	0.191	0.126–0.513	0.001^*^	0.036
CEBQ‐FR	0.278	0.252	0.152–0.404	< 0.0001^*^	0.063
CEBQ‐EOE	0.255	0.210	0.115–0.395	< 0.001^*^	0.044
CEBQ‐DD	0.136	0.115	0.002–0.273	0.048^*^	0.023
CEBQ‐SR	−0.415	−0.272	−0.588 to −0.243	< 0.001^*^	0.074
CEBQ‐SE	−0.360	−0.310	−0.490 to −0.230	< 0.001^*^	0.096
CEBQ‐EUE	−0.120	−0.097	−0.266 to 0.026	0.106	0.009
CEBQ‐FF	−0.054	−0.045	−0.194 to 0.087	0.452	0.002

*Note*: Linear regression analysis. Dependent continuous variable: BMI *Z*‐score.

Abbreviations: CEBQ‐DD, desire for drinks; CEBQ‐EF, enjoyment of food; CEBQ‐EOE, emotional overeating; CEBQ‐EUE, emotional undereating; CEBQ‐FF, food fussiness; CEBQ‐FR, food responsiveness; CEBQ‐SE, slowness in eating; CEBQ‐SR, satiety responsiveness; CI, confidence Interval;

* *p *< 0.05.

## 4 Discussion

The validation of the Spanish version of the CEBQ for school‐aged children provides valuable insights into its psychometric properties, enhancing its applicability across broader age groups. This study demonstrates that the CEBQ is a reliable tool for assessing eating behaviors in Spanish children aged 6–16 years, corroborating findings from previous validations in other populations and age groups.

The recent study by Jimeno‐Martínez et al. (2022) was the first to validate the Spanish‐translated version of the CEBQ in a population of 3‐ to 6‐year olds. The current study is the first to apply it to a Spanish sample aged 6–16 years. The results confirm that the Spanish version also exhibits strong psychometric attributes, including internal consistency, reliability, factor structure, and correlations between subscales. It is noteworthy that the CEBQ was initially designed for preschool‐aged children, although its use has been expanded in recent years to include school‐age and adolescent children (Hiremath et al. 2019; Czepczor‐Bernat and Brytek‐Matera 2019 ; Silva et al. 2020; Cho et al. 2022; Bjørklund et al. 2022). Consequently, this study employed the CEBQ for all schoolchildren aged 6–16 years. However, other researchers prefer using an adaptation of the Adult Eating Behavior Questionnaire (AEBQ) for this age group, also finding consistent results (Hunot‐Alexander et al. 2019; Molitor et al. 2021; Warkentin et al. 2022).

Our CFA indicated that the original 8‐factor structure of the CEBQ fits the data well, explaining 68.83% of the variance, a slightly higher proportion than reported in similar studies, such as Al‐Hamad et al. (2021) with Saudi preschoolers (60.17%) and Domoff et al. (2015) with U.S. preschoolers (62%). The RMSEA (0.066) and CFI (0.911) values also meet the strict criteria suggested by Hu and Bentler (1999) for model adequacy, reinforcing the robustness of the questionnaire structure. Comparatively, other studies have reported similar values. Notably, the study by Al‐Hamad et al. (2021) with a Saudi preschool population had an RMSEA of 0.055 and a CFI of 0.910, reflecting a comparable fit to our study. Likewise, Domoff et al. (2015) with U.S. preschoolers, reported an RMSEA of 0.050 and a CFI of 0.920, which align closely with acceptable standards, albeit slightly better in RMSEA. Finally, Svensson et al. (2011), in a Swedish population, reported an RMSEA of 0.045 and a CFI of 0.940, values slightly stronger than ours but still highly comparable.

The retention of items with cross‐loadings in their original subscales, as supported by studies like Gebru et al. (2021), ensures consistency across studies and improves comparability. In our analysis, cross‐loaded items were observed in “Enjoyment of Food” (EF), “Satiety Response” (SR), and “Reduced Emotional Eating” (EUE). These findings align with those of Gebru et al. (2021) in Ethiopian children aged 3–6 years and Jimeno‐Martínez et al. (2022) in Spanish children of the same age. However, the overlap observed between SR and “Slow Eating” (SE) aligns with reports by Carnell and Wardle (2007) and Sleddens et al. (2008), suggesting areas for refinement in future versions of the questionnaire to reduce redundancies and improve factorial clarity.

A critical comparison with other validation studies reveals both consistent and notable differences. For example, while the factor structure and reliability estimates in our study closely reflect those of the Swedish study by Svensson et al. (2011), our findings differ from the Ethiopian validation by Gebru et al. (2021), where some subscales required cultural adaptations. These variations highlight the importance of considering context and culture in psychometric evaluations.

Regarding the KMO, our value of 0.872 significantly exceeds the recommended minimum threshold of 0.60 and is similar to those reported in studies like Gebru et al. (2021) in Ethiopian children (KMO = 0.860) and Jimeno‐Martínez et al. (2022) in Spanish children aged 3–6 years (KMO = 0.850). This suggests a high adequacy of the data for factor analysis in our sample.

The Cronbach's alpha coefficients in this study reached acceptable values (*α* > 0.70) in most subscales, particularly high in “Food Responsiveness” (FR *α* = 0.879) and “Food Fussiness” (FF *α* = 0.839). These findings align with those reported by Viana et al. (2008) in Portugal (*α* = 0.85 for FR and *α* = 0.83 for FF) and Sleddens et al. (2008) in the Netherlands. However, the subscale “Enjoyment of Food” (EF) showed a lower value (*α* = 0.631) in our study, a finding that reflects limitations already reported by Jimeno‐Martínez et al. (2022) in younger Spanish children (*α* = 0.65). These discrepancies may be related to differences in item interpretation within each cultural context.

On the other hand, the correlations observed between subscales are consistent with previous studies, though some relationships warrant further analysis. For example, the strong positive correlation between “Emotional Overeating” (EOE) and “Food Responsiveness” (FR) (*γ* = 0.72 in our study) has been documented as indicative of uncontrolled eating behaviors in emotional contexts (Robinson et al. 2014). In contrast, the negative correlation between “Satiety Response” (SR) and “Enjoyment of Food” (EF) (*γ* = −0.65) reaffirms the dichotomy between pro‐ and anti‐eating behaviors, findings also reported by Silva et al. (2020) and Svensson et al. (2011).

In addition, studies like Viana et al. (2008) suggest that these correlations may vary based on cultural and socioeconomic factors, which could be explored in future cross‐sectional analyses in more diverse populations.

Regarding gender and age, our results indicate minimal gender differences, consistent with previous studies (Wardle et al. 2001; Svensson et al. 2011), where only small variations were observed in specific subscales, such as DD, where boys tend to score higher. However, age‐related differences are more pronounced. In this study, older children showed a significant decrease in FF, which may reflect greater exposure to a variety of foods and greater acceptance with age, a finding consistent with Silva et al. (2020) in Brazil. Similarly, the increase in EOE among older children suggests an emotional development that influences overeating in negative contexts, a pattern documented by Bjørklund et al. (2022) in Norway and by Domoff et al. (2015) in the United States.

Furthermore, studies like Robinson et al. (2014) highlight that these variations could be influenced by hormonal changes during puberty, impacting both food selectivity and emotional responses to food. These differences emphasize the need for personalized interventions that consider emotional and social development at each stage of growth, specifically addressing the needs of children more vulnerable to eating disorders.

This study presents certain limitations that should be discussed to contextualize the results. Despite its strengths, our study lacks test–retest reliability analysis, a crucial component for assessing temporal stability, as emphasized by Tsang et al. (2017). In addition, reliance on parent self‐reporting may introduce social desirability biases, which could be mitigated through direct observations or assessments in controlled settings, as suggested by Silva et al. (2020). The sample, while representative of the Madrid region (Spain), lacks cultural and socioeconomic diversity, limiting the generalization of the results. Finally, the cultural differences identified in studies such as Gebru et al. (2021) underline the need for specific adaptations to the questionnaire to optimize its cross‐cultural validity.

Future research should incorporate longitudinal designs to assess consistency over time and across different contexts. In addition, expanding the sample to include children from diverse socioeconomic and cultural backgrounds within Spain would improve the generalization of findings. These improvements will not only strengthen the validity of the CEBQ but also enable more precise interventions in pediatric obesity and behavioral nutrition.

## 5 Conclusions

Our study supports the use of the CEBQ as a psychometric tool for assessing eating behavior in Spanish schoolchildren aged 6–16 years. The behavior pattern observed differs minimally between genders but changes with age. The association between scores on the CEBQ's pro‐eating and anti‐eating subscales and BMI validates the questionnaire's utility in identifying specific eating traits associated with weight status. This suggests its value in obesity prevention interventions.

In conclusion, this study substantiates the validity and reliability of the CEBQ for assessing eating behaviors in Spanish children aged 6–16 years, with a solid factorial fit and consistent correlations with previous research. By filling gaps in the age range and providing comparative perspectives with prior validations, it highlights the CEBQ's usefulness as a robust tool for identifying behavioral traits linked to nutritional status and obesity risk in pediatric populations.

## Author Contributions

**Andrea Calderón García**: Conceptualization, investigation, writing–original draft, methodology, visualization, writing–review and editing, formal analysis, data curation, supervision. **Roberto Pedrero‐Tomé**: Investigation, methodology, validation, software, formal analysis. **Ana Alaminos‐Torres**: Conceptualization, investigation, supervision, writing–original draft, data curation. **Noemí López‐Ejeda**: Conceptualization, investigation, methodology, project administration, data curation. **María Sánchez Álvarez**: Conceptualization, investigation, methodology, data curation. **Consuelo Prado Martínez**: Supervision, validation, conceptualization, investigation. **Jesús Román Martínez Álvarez**: Conceptualization, investigation, funding acquisition, validation. **María Dolores Marrodán Serrano**: Funding acquisition, investigation, conceptualization, supervision, resources, project administration.

## Ethics Statement

The study was conducted in accordance with the Declaration of Helsinki and approved by The Ethics Committee of the AUTONOMOUS UNIVERSITY OF MADRID (CEI‐91‐1699‐2018)

## Consent

Informed consent was obtained from the parents or guardians of all subjects involved in the study.

## Conflicts of Interest

The authors declare no conflicts of interest.

### Peer Review

The peer review history for this article is available at https://publons.com/publon/10.1002/brb3.70343

## Supporting information



Table S1. Pro and anti‐intake dimensions, subscales, and corresponding questions in the assessment of the CEBQ questionnaire. Source: Wardle et al. 2001.
Table S2. Spearman Correlation between Scores of Different Scales of the CEBQ.

## Data Availability

The data for this study will be available upon request. The corresponding author has full access to the data reported in the manuscript.
