# Supplementary material for: Evaluation of the reliability and internal consistency of the child eating behavior questionnaire (CEBQ) in spanish schoolchildren and its association with obesity
Source: Brain Behav. 2025 Feb 19;15(2):e70343. doi: 10.1002/brb3.70343 (PMC11839750; doi:10.1002/brb3.70343)
Supplement: Supplementary file 1 — Table S1. Pro and anti‐intake dimensions, subscales, and corresponding questions in the assessment of the CEBQ questionnaire. Source: Wardle et al. 2001. Table S2. Spearman Correlation between Scores of Different Scales of the CEBQ. [file BRB3-15-e70343-s001.docx]

**Supplementary material**

**Table S1. Pro and anti-intake dimensions, subscales, and corresponding questions in the assessment of the CEBQ questionnaire. Source: Wardle et al. 2001.**

| **Prointake criterion** | **Antiintake criterion** |
| --- | --- |
| **Subscale 1: Enjoyment of Food (EF)**   1. Question 1: My child loves food. 2. Question 5: My child is interested in food. 3. Question 20: My child waits for meal times (breakfast, lunch...). 4. Question 22: My child enjoys eating. | **Subscale 5: Satiety Responsiveness (SR)**   1. Question 3: My child has a good appetite. 2. Question 17: My child leaves food on the plate after finishing the meal. 3. Question 21: My child gets full before finishing his/her meal. 4. Question 26: My child gets full easily. 5. Question 30: If my child has had something to drink before, he/she won't eat. |
| **Subscale 2: Food Responsiveness (FR)**   1. Question 12: My child is always asking for food. 2. Question 14: If allowed, my child would eat too much. 3. Question 19: Given the chance, my child would eat most of the time. 4. Question 28: Even if my child is full, there's always room for his/her favorite food. 5. Question 34: If given the opportunity, my child always has food in his/her mouth. | **Subscale 6: Slowness in Eating (SE)**   1. Question 4: My child finishes his/her meal quickly.* 2. Question 8: My child eats slowly. 3. Question 18: My child takes more than 30 minutes to finish a meal. 4. Question 35: My child eats more and more slowly during the course of the meal. |
| **Subscale 3: Emotional Overeating (EOE)**   1. Question 2: My child eats more when he/she is worried. 2. Question 13: My child eats more when he/she is bored. 3. Question 15: My child eats more when he/she is anxious. 4. Question 27: My child eats more when he/she has nothing to do. | **Subscale 7: Emotional Undereating (EUE)**   1. Question 9: My child eats less when he/she is angry. 2. Question 11: My child eats less when he/she is tired. 3. Question 23: My child eats more when he/she is happy. 4. Question 25: My child eats less when he/she is angry. |
| **Subscale 4: Desire for Drinks (DD)**   1. Question 6: My child asks for liquids all the time. 2. Question 29: If given the chance, my child would drink constantly throughout the day. 3. Question 31: If given the chance, my child would always be drinking something. | **Subscale 8: Food Fussiness (FF)**   1. Question 7: My child initially refuses new foods. 2. Question 10: My child enjoys tasting new foods.* 3. Question 16: My child enjoys a variety of foods.* 4. Question 24: My child is hard to please with meals. 5. Question 32: My child is interested in trying foods he/she has not tasted before.* 6. Question 33: My child decides he/she doesn't like a food even without trying it. |

***Table S2. Spearman Correlation between Scores of Different Scales of the CEBQ.***

|  | **CEBQ-**  **EF** | **CEBQ-**  **FR** | **CEBQ-**  **EOE** | **CEBQ-**  **DD** | **CEBQ-**  **SR** | **CEBQ-**  **SE** | **CEBQ**  **EUE** | **CEBQ-**  **FF** |
| --- | --- | --- | --- | --- | --- | --- | --- | --- |
| **CEBQ-**  **EF** | 1 |  |  |  |  |  |  |  |
| **CEBQ-**  **FR** | 0.267** | 1 |  |  |  |  |  |  |
| **CEBQ-**  **EOE** | 0.092 | 0.670** | 1 |  |  |  |  |  |
| **CEBQ-**  **DD** | 0.052 | 0.424** | 0.353** | 1 |  |  |  |  |
| **CEBQ-**  **SR** | -0.619** | -0.353** | -0.166** | -0.003 | 1 |  |  |  |
| **CEBQ-**  **SE** | -0.493** | -0.298** | -0.162** | 0.004 | 0.656** | 1 |  |  |
| **CEBQ-**  **EUE** | -0.128* | 0.160** | 0.475** | 0.236** | 0.287** | 0.210** | 1 |  |
| **CEBQ-**  **FF** | -0.557** | -0.118* | 0.004 | -0.005 | 0.416** | 0.289** | 0.214** | 1 |

CEBQ-EF: Enjoyment of food. CEBQ-FR: Food responsiveness. CEBQ- EOE: Emotional overeating. CEBQ-DD: Desire for drinks. CEBQ-SR: Satiety responsiveness. CEBQ-SE: Slowness in eating. CEBQ-EUE: Emotional undereating. CEBQ-FF: Food fussiness. Spearman Correlation Analysis between Different Subscales. Significant differences * p < 0.05; **p < 0.001.
